# What does the wolf eat? Assessing the diet of the endangered Iberian wolf (*Canis lupus signatus*) in northeast Portugal

**DOI:** 10.1371/journal.pone.0230433

**Published:** 2020-03-31

**Authors:** Ana M. Figueiredo, Ana M. Valente, Tânia Barros, João Carvalho, Davide A. M. Silva, Carlos Fonseca, Luís Madeira de Carvalho, Rita Tinoco Torres

**Affiliations:** 1 Department of Biology & CESAM, University of Aveiro, Aveiro, Portugal; 2 Instituto de Investigación en Recursos Cinegéticos (UCLM-CSIC-JCCM), Ciudad Real, Spain; 3 CIISA—Centre for Interdisciplinary Research in Animal Health, Faculty of Veterinary Medicine, University of Lisbon, Lisbon, Portugal; Universitat Autonoma de Barcelona, SPAIN

## Abstract

The Iberian wolf (*Canis lupus signatus*) is a top predator that inhabits the Iberian Peninsula. In Portugal, its numbers and distribution declined throughout the 20^th^ century, due to human persecution, habitat degradation and prey decline, which have led to higher predation rates of livestock in the remaining packs. In Montesinho Natural Park (northeast Portugal), wild ungulate populations have been increasing in the last years, which may have led wolf to predate upon them. In order to assess Iberian wolf diet in this area, 85 wolf scats were collected from transects distributed throughout the study area in two periods between November 2017 and August 2019. Scat analysis indicated a high predation on wild ungulates, where the frequency of occurrence showed that roe deer was the most consumed prey (44%), followed by red deer (26%) and wild boar (24%). Domestic/wild cat (6%), domestic goat and stone marten (5%) were consumed in lower quantities. It was found a higher selection towards roe deer (D = 0.71) and this was the only prey item which was significantly dependent of the season of the year (χ^2^ = 16.95, df = 3, p < 0.001). This is the first study in Portugal where was recorded that wolves feed mainly on wild ungulates. We conclude that lower livestock predation may be correlated with higher wild ungulates densities in our study area, as well as suitable husbandry practices, leading to a shift on Iberian wolf diet from mainly livestock on previous studies to wild ungulates.

## Introduction

The grey wolf (*Canis lupus*) is considered one of the world’s most widespread mammal [1]. As an apex predator, this species can contribute to restore local biodiversity and trophic interactions, which ultimately leads to ecosystem recovery [1], [2]. In Europe, wolf original range was drastically reduced in the end of the 19^th^ century, mainly due to human persecution, habitat degradation and prey decline [3], being eradicated from most central and northern countries [4], [5], [6]. The rooted conflict between this predator and humans is mostly due to livestock predation [7]. This is mostly aggravated in areas where wild prey diversity and density is low [6]. However, in the last decades, due to legal protection policies [4], natural recolonization [8] and wild ungulate increase [9], [10], wolf populations have recovered and are now expanding their ranges across some countries in Europe [1], [4], [5], [11].

In the Iberian Peninsula inhabits an endemic subspecies of the grey wolf, the Iberian wolf (*Canis lupus signatus*). However, in Portugal, contrasting to the European scenario, this species declined dramatically during the 20^th^ century, both in number and distribution, gradually disappearing from coastal, south and central regions of the country [3], [6], [12]. Facing this fast decline, since 1988 this species is protected by law and listed as “Endangered” in the Portuguese Red Data Book of Vertebrates [3], [13].

Although the wolf is recognized as a generalist predator, its feeding ecology differs across Europe and seems to be mostly related with wild prey abundance [14], age and physical condition of the available prey [15] and livestock protection measures [6], [9], [16], [17]. In central and northern Europe, due to the high abundance of wild prey (*e*.*g*. red deer *Cervus elaphus*, roe deer *Capreolus capreolus* and wild boar *Sus scrofa*), wolves’ diet is mainly based on these species [7], [18], [19], [20], [21], [22], [23]. In south Europe, however, lower wild prey densities and human-dominated landscapes have led wolves to prey upon livestock and even human garbage [9], [16], [17], [24] [25].

In Portugal, studies focused on wolf feeding ecology reveal higher preference for livestock, either on north and south of Douro River [6], [12], [16], [26], [27]. Torres et al [6] showed that livestock made up over 90% of this predator diet. However, increasing numbers and expansion of wild ungulates in the last decades across Portugal, either from natural re-colonization or reintroductions [10], [28], [29], [30] may have led to a shift on Iberian wolf diet, especially at northeast Portugal, where wild ungulate densities are relatively high [31], [32]. It is therefore timely to assess this endangered predator diet in northeast Portugal, particularly when the last record goes back to the 1970s [26], and has been later analysed only in the Spanish part of the same population [33], [34]. To fill this gap, the main aim of this study was to evaluate the Iberian wolf diet in northeast Portugal, comparing with the only available study performed in this area in 1978 [26]. Given that in Paixão de Magalhães and Petrucci-Fonseca [26] study wolf diet was mainly composed by livestock (52.8%) and considering the increasing densities of wild ungulates in northeast Portugal, we hypothesize that wolf feeding habits may have changed from mostly livestock to wild ungulates.

## Material and methods

### Ethics statement

Our research did not involve capture, handling or killing of animals, therefore did not require approval of animal care and use procedures. Permissions for field studies were given by Nature and Forestry Conservation Institute.

### Study area

**Fig 1:**
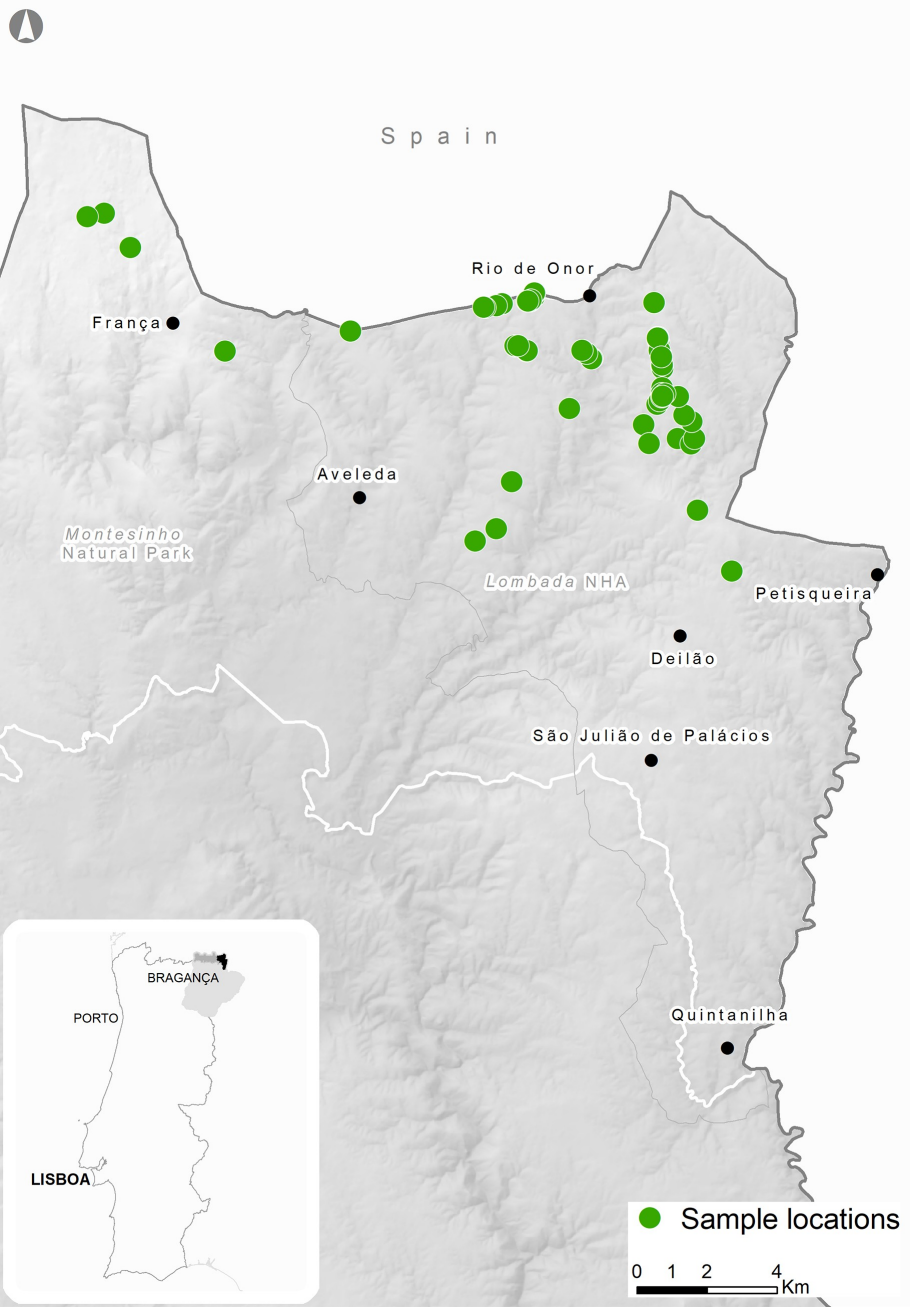
Location of the study area in Portugal. The green circles correspond to the location of Iberian wolf scats used in diet analysis (Montesinho Natural Park).

Our study was conducted in Montesinho Natural Park (MNP) (6°30’-7°12’W, 41°43’-41°59’N), comprised as one of European Union’s Natura 2000 Network sites (Fig 1). The total prospected area was 35,000 ha and is characterized by a mountainous landscape, with elevation ranging from 438 to 1,481 m. Our study area experiences a Mediterranean climate, with an annual average temperature ranging between 3°C in the coldest month and 21°C in the warmest, and precipitation between 600 and 1,500mm [35]. The area exhibits a mosaic of deciduous and coniferous forest, characterized by oaks (*Quercus pyrenaica*, *Q*. *rotundifolia*, *Q*. *suber*), sweet chestnut (*Castanea sativa*) and maritime pine (*Pinus pinaster*); shrub vegetation, dominated by heather (*Erica* spp.), gum rockrose (*Cistus ladanifer*) and furzes (*Ulex europaeus* and U. minor), and fragmented by small cultivated fields [31], [32]. Roe deer densities estimation is 1.23 ind./100 ha [31] and red deer 5.81 ind./100 ha [32]. Livestock density estimation throughout the study area is 0.54 ind./100 ha cattle, 0.01 ind./100 ha domestic pigs, 9.21 ind./100 ha sheep and 0.93 ind./100 ha goats [36], [DGAV, 2017, personal communication].

### Scat collection and laboratory analysis

Experienced and field-trained personnel collected wolf scats between November 2017 and August 2018 and between June and August 2019, throughout our study area (Fig 1). Paths, dirt roads, firebreaks, forest trails and crossroads were prospected using a vehicle (< 10km/h) or by foot. Morphology, size, scent, colour, contents and spatial position were used to identify wolf scats. Scats collected along the trails were stored in plastic bags, labelled and registered using a Global Positioning System (GPS) [6]. The collected scats were submitted to genetic analysis to confirm the species *Canis lupus signatus*, avoiding misclassification of domestic dog (*Canis lupus familiaris*) and red fox (*Vulpes vulpes*). DNA extraction was performed using QIAamp® DNA Stool Mini Kit (QIAGEN Hilden, Germany) following manufacturer instructions. A fragment of 350bp from the control region (mitochondrial region) [37], was amplified using the universal primers Thr-L 15926 5’- CAATTCCCCGGTCTTGTAAACC-3′ and DL-H 16340 5′-CCTGAAGTAGGAACCAGATG-3′ [36]. PCR mix reaction was performed with 2.5 μL of BSA, 0.85 μL of MgCl2, 0.5 μL of dNTPs, 0.3 μL of each primer (Thr-L and DL-H), 0.2 μL of Taq and 12.88 μL of double-distilled H2O, and then 5μL extracted DNA was added to the mix. Reaction mixtures were initially denatured at 94°C for 3 min, followed by 42 amplification cycles (94°C for 1 min; annealing for 2 min at 50° and extension for 1,5 min at 72°C) and a final extension step at 72°C for 10min (adapted from [38], [39]). Samples were visualized by electrophoresis on 1.4% agarose gel. Mitochondrial fragments were purified using ExoSap-IT® (USB Corporation) and sent to sequence in both directions using sequencers ABIPRISM® 3730-XL DNA Analyser from Applied BiosystemsTM. Sequences were then manually aligned using MEGA version 6.0 [40] and compared with previously published *Canis lupus signatus* sequences [37]. Since some scats were older, we were only able to extract DNA for wolf confirmation from 50% of them. Of that 50%, only 2.2% were excluded from belonging to red fox.

Regarding hair identification procedures, we followed Teenrik et al [41], De Marinis & Asprea [42] and Valente et al [43] protocols in order to prepare the hair slides for further identification. Wolf scats were firstly washed with water and examined macroscopically in order to differentiate hair from bones, feathers, vegetable and mineral material, insects and garbage. After drying, hair slides were prepared, and the consumed prey items were identified through microscopic examination of their cuticular pattern, medulla and cross-section [6].

### Wolf diet analysis

To evaluate wolf diet based on hair identification, we used frequency of occurrence (FO) as a measure to quantify prey items. FO is considered the most common method used in diet analysis; however, it may overestimate the frequency of difference size preys, taking into account their ratio surface/volume (preys either with bigger body mass or small ones) [6], [44], [45]. For each prey item, FO was categorized according to Ruprecht [46]: basic food (≥20%); regular food (5–20%); supplementary food (1–5%) and sporadic food (≤1%). A χ^2^-test (significance level ρ≤0.05) was performed to assess differences in the FO of each prey item. The analyses were performed using the R software [47].

FO was then converted into the percentage of biomass of prey consumed, using the linear regression of Floyd et al [41], modified by Weaver [48]:

y=0.439+0.008*x

Where *y* = biomass consumed per scat

*x* = average weight (kg) of each prey identified in the scats.

Each y was then multiplied by the actual number of scats containing each prey type in order to estimate the total amount of biomass for each prey class.

Average body masses of the following prey species were estimated based on the literature: red deer (100kg), roe deer (24kg), wild boar (67kg), stone marten (1.7kg), domestic/wild cat (4.75kg), small mammals (0.02kg) and domestic goat (28.5kg) [18], [27], [49], [50], [51], [52], [53], [54].

Levins’ index of niche breadth [55] was calculated according to the following formula:

$$B=\frac{1}{\sum p_{j}^{2}}$$

Where B = Levins’ measure of niche breadth

p_j_ = proportion of prey items from food category j

Levins’ measure was then standardized on a scale of 0 (specialist predator–strong specialization in one group of prey) to 1 (generalist predator–opportunistic preying on all groups of preys), according to Hurlbert’s formula [56]:

$$B_{A}=\frac{B-1}{n-1}$$

Where B_A_ = Levins’ standardized Food Niche Breadth

B = Levins’ Food Niche Breadth

*n* = number of prey items found in the diet

Shannon’s Diversity Index (H’) was calculated in order to obtained diet evenness [57], [58]:

$$H^{\prime }=-\sum p_{i}*ln(p_{i})$$

Where p_j_ = proportion of prey species i in the diet

Diet evenness was standardized on a scale of 0 (uneven) to 1 (complete evenness), following Shannon equitability diversity (Eh) formula:

$${Eh}_{}=\frac{H\prime }{H\prime max}$$

Where *Eh* = Shannon equitability diversity

H’ = Shannon’s Diversity Index

H’max = ln(S) where S represents the total number of prey items

The last index calculated was Ivlev’s electivity index (D) [59], modified by Jacobs [60], to measure wolf prey preference from -1 (total avoidance of a species) through 0 (no selection) to +1 (maximum positive selection):

$$D=\frac{\left(r_{i}-p_{i}\right)}{\left(r_{i}+p_{i}-2r_{i}p_{i}\right)}$$

Where *r* = proportion of a given prey species in wolf diet

p = proportion in the free-living population

Roe deer and red deer proportion was calculated based on Valente et al [31] and Torres et al [32] density estimation. Domestic goat community proportion was estimated using data from the National Institute of Statistics [36] for the study area. The Ivlev’s electivity index could not be estimated for wild boar because there was no available data from monitoring studies or official game inventories for the study area, as well as for stone marten, small mammals and domestic/wild cat.

Both Levins’ standardized Food Niche Breadth (B_A_) and Shannon equitability diversity (Eh) were calculated for Paixão de Magalhães & Petrucci-Fonseca [26], using the FO of the prey items found in their study for further comparison with our indices. Ivlev’s electivity index (D) was not possible to estimate because we did not have access to prey density data for both livestock/wild species in the previous study.

Seasonal variation of the main prey species found in wolf diet was calculated using a χ^2^-test, defining a significance level of ρ ≤0.05. We used the χ^2^-test to understand if (1) the FO is different for each season (Autumn, Winter, Spring and Summer); and (2) the FO of each individual prey item (red deer, roe deer, wild boar, domestic/wild cat, stone marten, domestic goat and small mammals) are seasonally dependent.

## Results

**Fig 2:**
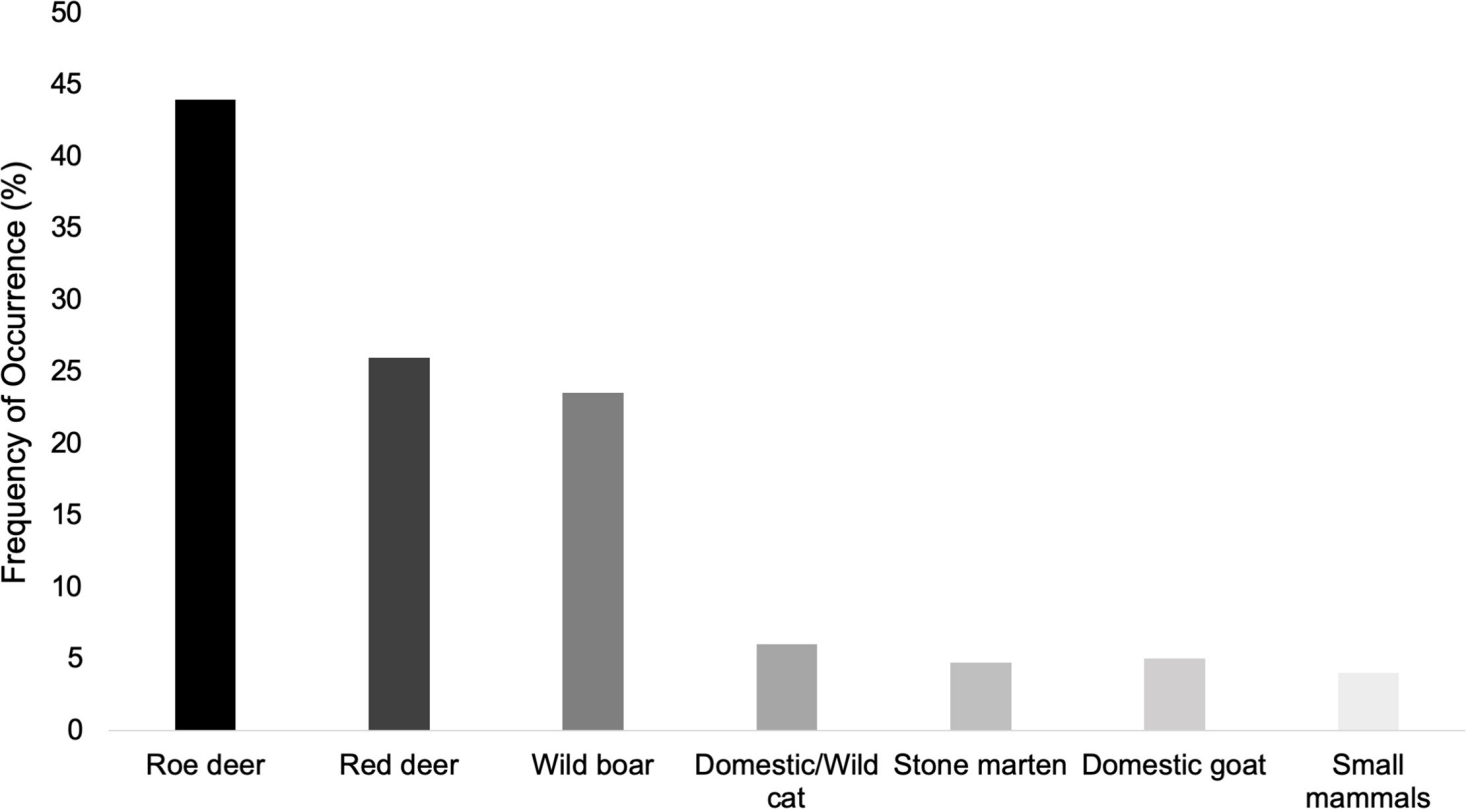
Composition of Iberian wolf diet in terms of frequency of occurrence (FO) in northeast Portugal, Montesinho Natural Park (MNP).

Between November 2017 and August 2019, a total of 85 wolf scats were collected throughout the study area. Seven different prey items (wild boar, red deer, roe deer, domestic goat, domestic/wild cat, stone marten and small mammals) were identified (see supplementary information). Fig 2 shows diet composition expressed in FO of prey remains in scats. In total, 87% of all analysed scats presented only one prey item, whereas in 13% were found two prey items. According to the FO, roe deer was the most consumed prey (44%), followed by red deer (26%) and wild boar (24%), categorized as basic food items on wolf’s diet. Domestic/wild cat (6%), domestic goat and stone marten (5%) were also found in wolf’s diet, although in lower percentage as regular food items, while small mammals were the least consumed prey (4%), being categorized as supplementary food item [46]. The χ^2^-test showed significant differences in the FO of each prey item in the total analysed samples (χ^2^ = 76.38, df = 6, p<0.001).

**Fig 3:**
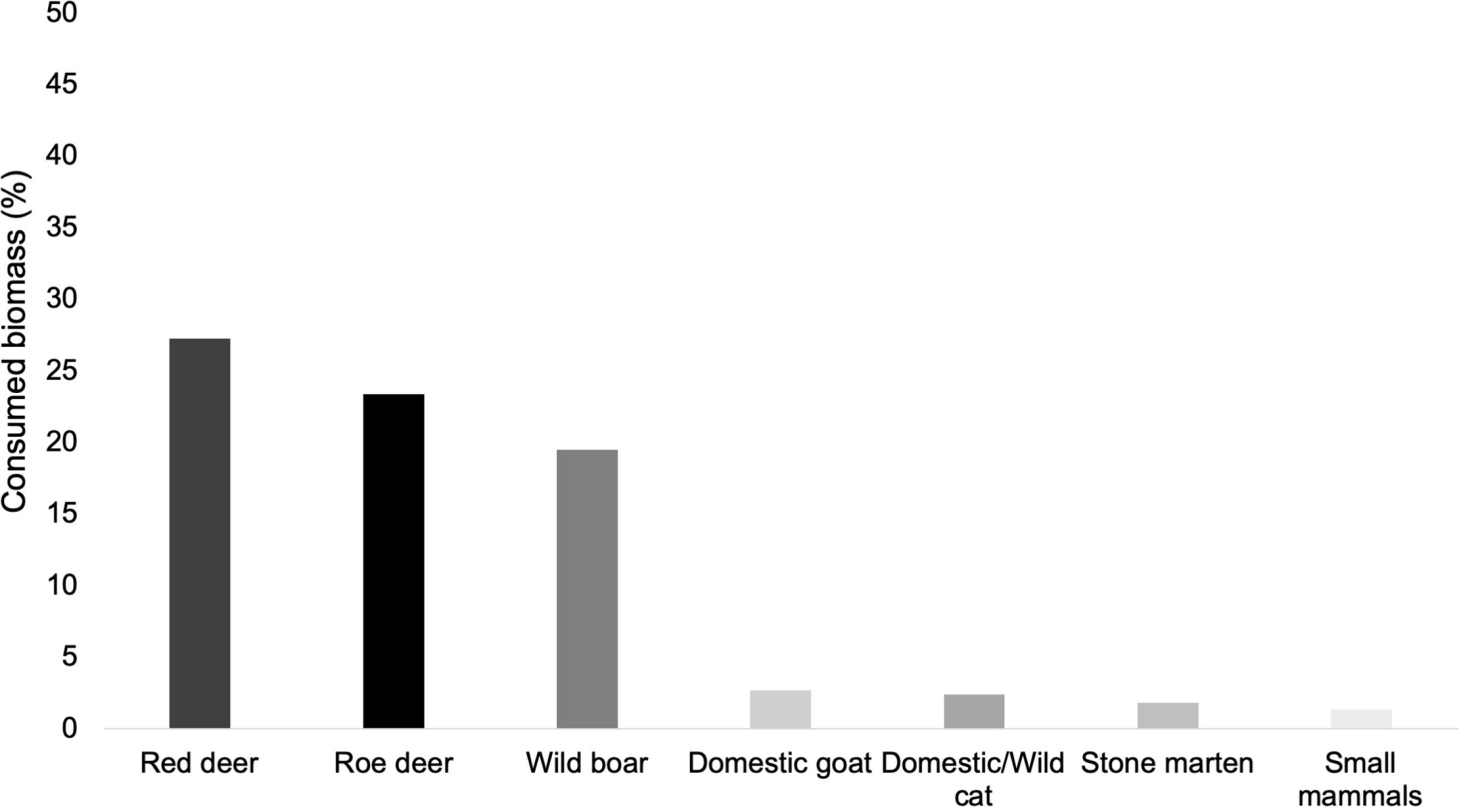
Biomass consumed from each prey species identified in Iberian wolf diet in northeast Portugal, Montesinho Natural Park (MNP).

When considering prey consumed biomass, red deer was the most consumed prey (27.3%), followed by roe deer (23.3%) and then wild boar (19.5%) (Fig 3). For both FO and consumed biomass, wild ungulates were the most consumed prey of wolf’s diet (83%), and both domestic goat and all the other wild preys found on the scats represent only a small fraction of its diet.

**Fig 4:**
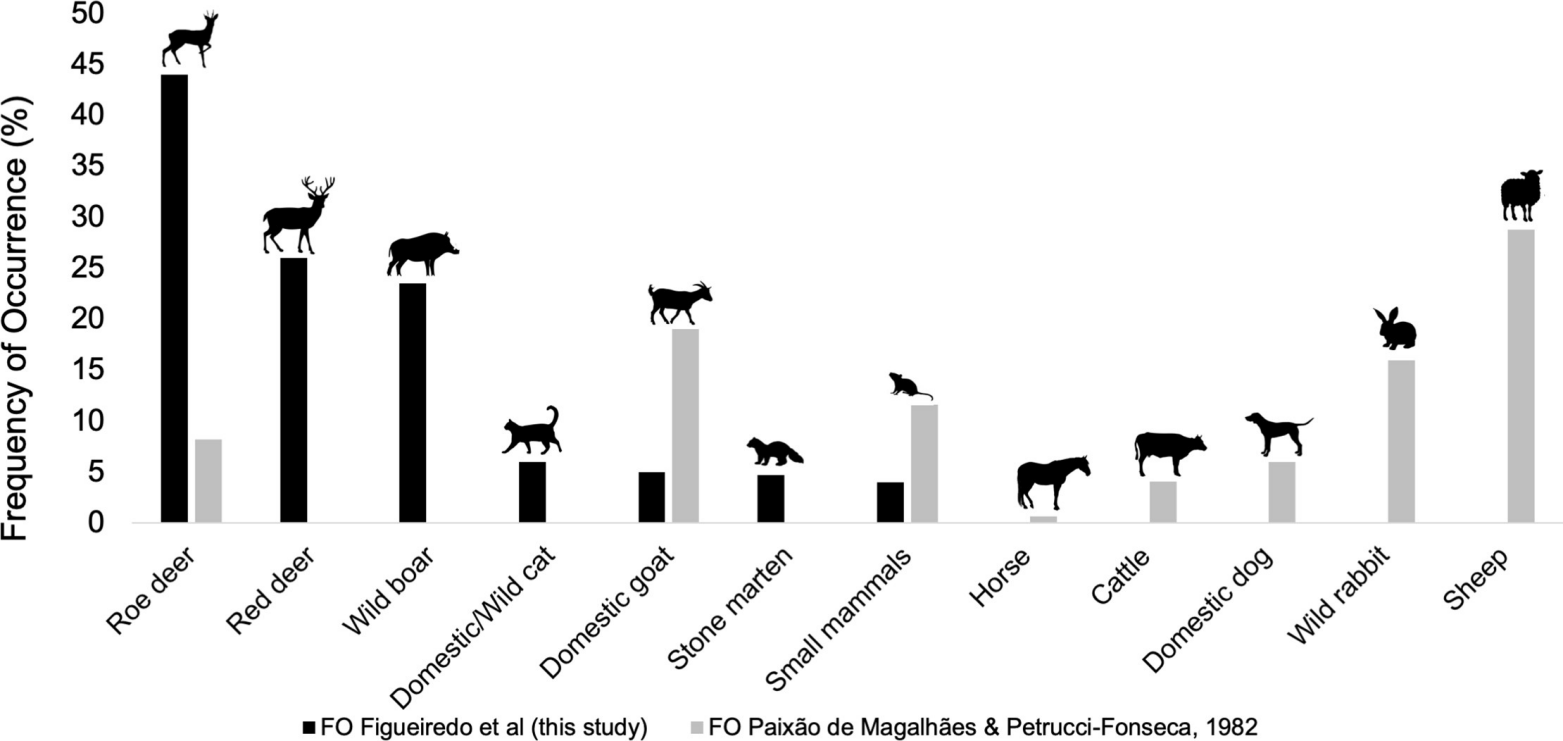
Comparison of Iberian wolf diet in terms of frequency of occurrence (FO) for Paixão de Magalhães & Petrucci-Fonseca (1982) [26] and this present study in northeast Portugal, Montesinho Natural Park (MNP).

Fig 4 shows a visual comparison of the FO found in Paixão de Magalhães and Petrucci-Fonseca [26] study and the one found in this study.

Niche breadth index for this study, estimated by the standardized Levin’s index was closest to zero (B = 0.35), indicating a tendency towards a more specialist feeding habit. Shannon equitability diversity indicated a higher evenness, suggesting that each identified prey item is almost equally consumed by the Iberian wolf (Eh = 0.83). On the other hand, Levin’s index for Paixão de Magalhães and Petrucci-Fonseca [26] study was closest to 1 (B = 0.69), indicating a tendency towards a more generalist feeding habit, while Shannon equitability diversity indicated a higher evenness, practically equal to the one found in the present study (Eh = 0.85).

**Fig 5:**
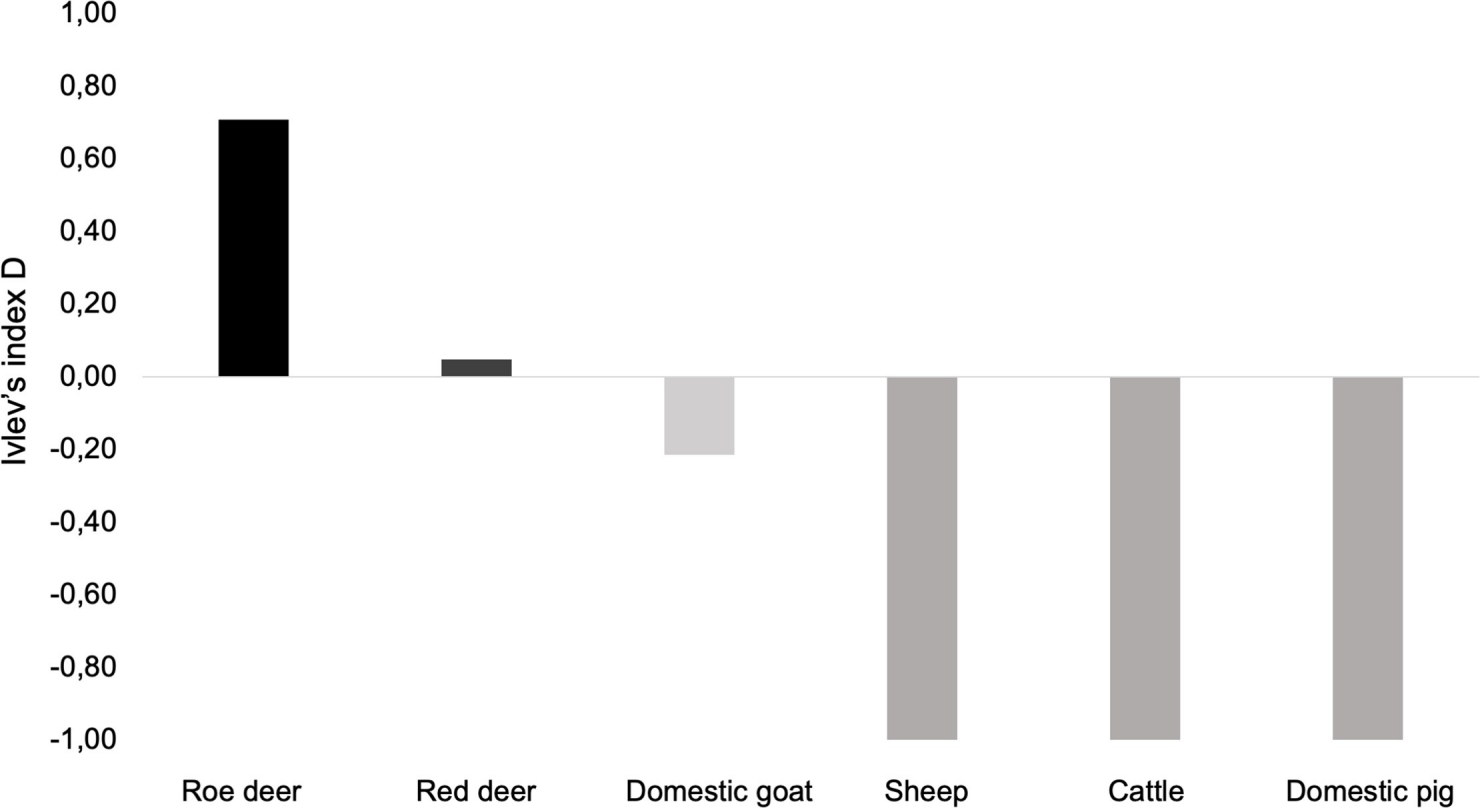
Prey selectivity (Ivlev’s index D) calculated for roe deer *Capreolus capreolus*, red deer *Cervus elaphus*, domestic goat *Capra hircus*, sheep *Ovis aries*, cattle *Bos taurus* and domestic pig *Sus scrofa domesticus* based on Iberian wolf scat analysis (n = 85) in Montesinho Natural Park, northeast Portugal. The wolf selects species with positive index values, while species with negative index are negatively selected.

Ivlev’s electivity index calculated for this study (Fig 5) showed a higher selection towards roe deer (D = 0.71), and almost no selection towards red deer (D = 0.05). Domestic goat was negatively selected, taking into account their availability in the study area, meaning that it is consumed less than expected by their availability (D = -0.21). Considering sheep, cattle and domestic pig availability and densities in the study area and given that these species were never identified in the analysed samples, Ivlev’s index showed that wolf never select any of these species (D = -1.00).

**Fig 6:**
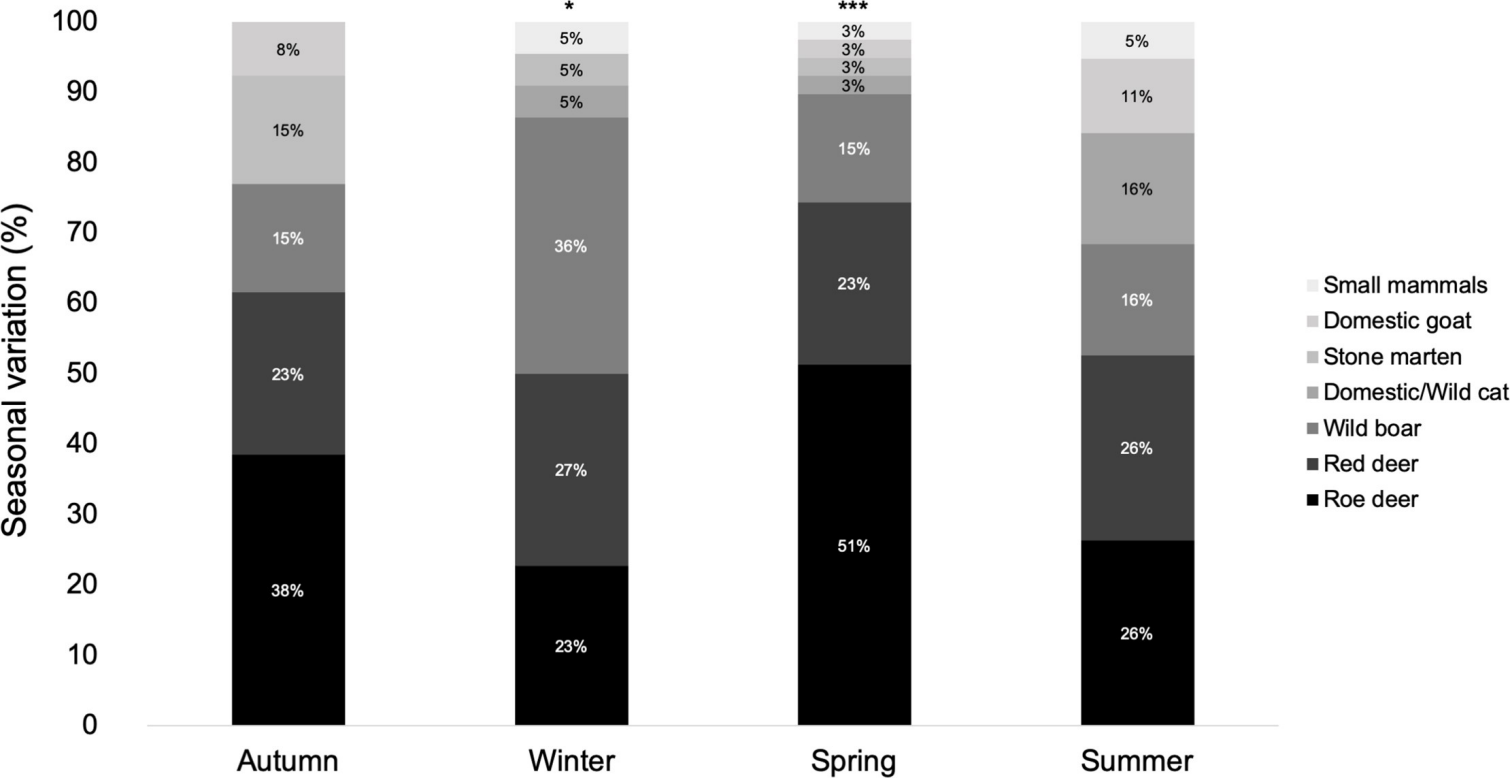
Seasonal variation of Iberian wolf diet in Montesinho Natural Park, northeast Portugal. Significant differences in the FO of the prey items were identified in Winter (*) (χ^2^ = 12.91, df = 5, p = 0.02) and Spring (***) χ^2^ = 54.51, df = 6, p = 5.81 x 10^−10^).

Seasonal variation, expressed in FO, demonstrated that wild ungulates represent the main prey items on wolf’s diet during all seasons, with values ranging from 68% in Summer and 89% in Spring (Fig 6). All seven prey items found in the collected scats were only consumed in Spring, while Autumn was the season with less prey diversity. Roe deer was the most consumed prey in both Spring and Autumn (51% and 38%, respectively), being equally consumed as the red deer in Summer (26%) and slightly less consumed than the red deer (27%) in Winter (23%). χ^2^-test analysis revealed that (1) the FO is significantly different for Winter (χ^2^ = 12.91, df = 5, p = 0.02) and Spring (χ^2^ = 54.51, df = 6, p<0.001) but not for Autumn and Summer; and (2) the only prey dependent of the seasons was the roe deer (χ^2^ = 16.95, df = 3, p<0.001).

## Discussion

In the Iberian Peninsula, the Iberian wolf shows a large spectrum of diet (livestock [6], [16], [27]; wild ungulates [25], [34], [61]). Our study showed that wild ungulates are the basis of wolves’ diet in MNP, Bragança. These results contrast with Paixão de Magalhães and Petrucci-Fonseca [26], where livestock made up 52.8% of the wolf’s diet, and wild ungulates only 8.2%. Fig 4 shows a visual representation of the Iberian wolf diet in terms of the FO found in Paixão de Magalhães and Petrucci-Fonseca [26] and in our study, where it is observed a clear shift on wolf’s feeding habits over the last decades, from mainly livestock (*e*.*g*. sheep (28.8%), domestic goat (19.2%)) to wild ungulates. At that time, low densities of roe deer were reported, and the red deer population was just starting to settle due to the natural expansion from the border Spanish population [62]. Other studies in Portugal showed that livestock make up most of the wolf diet, even at different rates. For example, Vos [16] found a prevalence of 97.5% for domestic goat in south of Douro river population, but in Peneda-Gerês (northern Portugal), goat made up 58.7% of wolf diet. Roque et al [27] and Álvares [12] showed that in the northwest population, livestock was the most consumed prey (84.7% and 81.7%, respectively), while wild ungulates were far less consumed (7.2% and 10.8%, respectively). The same trend was reported by Torres et al [6] for wolf packs located at south of Douro river. (>94% of livestock for the three packs evaluated). So, based on previous studies, wolves’ diet was mostly focused on livestock and, to the best of our knowledge, this is the first study in Portugal where wolves feed mainly on wild ungulates. The northeast Portugal is a particular case, where wild ungulate densities and diversity are nowadays relatively high [31], [32]. A similar trend has been observed in the Spanish side of the same wolf population [25], [33], [34], [63]. Cuesta et al [33] found in three out of five analysed areas, which cover most of the Iberian wolf’s Spanish distribution that wolves feed mostly upon wild ungulates. Similar results were found by Barja [34] were roe deer was considered the most important prey species in Iberian wolf diet in north-western Spain (see also Lagos & Bárcena [61]).

Seasonal variation showed significant differences for Winter and Spring season, and roe deer was the only prey significantly dependent of the seasons. These results can be explained due to spring births of the roe deer litter, which are an easy prey for the Iberian wolf [64]. Likewise, Paixão de Magalhães and Petrucci-Fonseca [26] reported higher FO of roe deer in spring due new-born birth. High availability and predation of juvenile roe deer, particularly in Spring and Summer, was also described by Barja [34], which is critical during the cub’s period, where a higher demand of energy is required.

Several studies in central and northern Europe have already reported a preference of wolf towards wild ungulates, due to higher density and diversity of wild preys [7], [22], contrasting with some southern Europe countries [6], [17]. Our results show that the larger percentage of consumed biomass was from red deer (27.3%), which is consistent with other European studies, although they found higher percentages: 42% [18], 43% [19], 59% [63], and 59.9% [65]. Likewise, other studies performed in northern Europe reported higher predation rates of roe deer (53.3%, [22]) and wild boar (35.6%, [21]), and that happened even in north Italy (wild boar, 58.9%, [45] and red deer, 28% [66]). According to Huggard [67], prey selectivity is dependent on habitat overlap, vulnerability and probability of prey occurrence and encounter rates between predator and prey. Across Europe, studies showed that roe deer was positively selected, and both red deer and wild boar were negatively selected [19], [22], which is consistent with our results. Wild boar is generally avoided by wolves in Europe [68], however can be positively selected if there is lower abundance of both red and roe deer [18], [69]. Although wolves main prey items are ungulates (either wild or domestic), they are considered generalist carnivores, having a significant diet breadth and feeding from different species throughout their range [70], [71]. Our results show that wolves tend to have a more specialist diet towards wild ungulates, however Shannon’s diversity index indicates evenness, meaning that each prey item found in our study have a tendency to be equally consumed by wolves. Wolf selection of a given prey may be dependent not only on the relative abundance of that species, but also on alternative prey availability [66]. Livestock species are the most consumed item by the Portuguese wolf packs, which can be explained by the low abundance and diversity of wild ungulates, either at south of Douro River packs [6], [16], and at northwest packs [12], [16], [27]. In Paixão de Magalhães and Petrucci-Fonseca [26] study, low abundance and diversity of wild ungulates, lead wolves to acquire a more generalist feeding habitat, and Shannon’s diversity index also indicates evenness on prey consumption. Since 1978 [26], besides the increasing wild prey availability in MNP, improving husbandry practices play a major role in explaining the current lower rates of livestock predation found in our study area, since shepherds and guarding dogs are always seen during daylight, escorting cattle and small ruminants, which is considered one of the most effective ways of protecting livestock from wolf predation [17]. Although semi-confined farming systems are dominant in our study area, guarding dogs are essential, especially at night, in order to reduce wolf attacks and costs of compensation schemes [71], but also human-wildlife conflicts [72]. Our study shows a significant domestic goat avoidance, which may be related with improved livestock protection measures, which consequently reduces the predation. Pimenta et al [73] predicted wolf predation probability for our study area, where sheep was the livestock species with higher predation risk, while goat was the second and donkeys the third. However, Pimenta et al [73] results are correlated with livestock densities estimated for our study area, where sheep was the species with higher density (9.211 ind/100 ha) (DGAV, 2017, personal communication). Our results did not support this hypothesis.

Stone marten and domestic/wild cats were found with the same frequency of occurrence of domestic goat on wolf’s diet. Carnivore’s consumption by wolves can be correlated with interspecific interactions, which may be associated with spatial and trophic competition but also as an important additional food source [12], [74]. It is important to stress that scat analysis, through hair identification, only reveals what wolves ate and that does not necessarily have to correspond to what they killed, since scavenging events can occur. Yet, as the wolf is the only top predator in the study area, there are no reasons to believe that wild ungulate presence in the wolf diet does not reflect wolf predation.

## Conclusion

Our results showed that Iberian wolf diet in northeast Portugal is mainly composed by wild ungulates, contrasting with studies performed in other Portuguese packs. These results can be explained by both a decrease in the number of head of cattle since the 80's and 90's and an increase of wild ungulates densities in our study area in the last decades, in comparison with other areas in Portugal. The presence of wild preys can be envisioned as the most welcome step towards the mitigation of human-predator conflicts, contributing to the conservation of the Iberian wolf in Portugal.

## Supporting information

S1 File (DOCX)

## References

[1] Ripple W. J., Estes J. A., Beschta R. L., Wilmers C. C., Ritchie E. G., Hebblewhite M., et al Status and ecological effects of the world’s largest carnivores. Science. 2014, 343(6167), 1241484 10.1126/science.1241484 24408439

[2] Boyce M. S. Wolves for Yellowstone: dynamics in time and space. J Mammal. 2018, 99(5), pp. 1021–1031.

[3] Torres R. T., & Fonseca C. Perspectives on the Iberian wolf in Portugal: population trends and conservation threats. Biodivers Conserv. 2016, 25(3), pp. 411–425.

[4] Boitani L. Action plan for the conservation of wolves in Europe (*Canis lupus*) (No. 18–113). Council of Europe Publishing, 2000.

[5] Eggermann J., da Costa G. F., Guerra A. M., Kirchner, et al Presence of Iberian wolf (*Canis lupus signatus*) in relation to land cover, livestock and human influence in Portugal. Mamm Biol. 2011, 76(2), pp. 217–221.

[6] Torres R. T., Silva N., Brotas G., & Fonseca C. To eat or not to eat? The diet of the endangered Iberian wolf (*Canis lupus signatus*) in a human-dominated landscape in central Portugal. PloS one, 2015 10(6), e0129379 10.1371/journal.pone.0129379 26030294 PMC4452520

[7] Zlatanova D., Ahmed A., Valasseva A., & Genov P. Adaptive diet strategy of the wolf (*Canis lupus* L.) in Europe: a review. Acta Zool Bulgar. 2014, 66(4), pp. 439–452.

[8] Marucco F., & McIntire E. J. B. Predicting spatio‐temporal recolonization of large carnivore populations and livestock depredation risk: wolves in the Italian Alps. J Appl Ecol. 2010, 47(4), pp. 789–798.

[9] Meriggi A., & Lovari S. A review of wolf predation in southern Europe: does the wolf prefer wild prey to livestock? J Appl Ecol. 1996, pp. 1561–1571.

[10] Torres R. T., Brotas G., Fonseca C. Roe deer reintroduction in central Portugal: a tool for Iberian wolf conservation. Global Reintroduction Perspectives: 2018. Case studies from around the globe, 139 2018.

[11] Chapron G., Kaczensky P., Linnell J.D., von Arx M., Huber D., Andrén H., et al Recovery of large carnivores in Europe’s modern human-dominated landscapes. *Science*. 2014, 346(6216), pp.1517–1519. 10.1126/science.1257553 25525247

[12] Álvares, F. Ecologia e conservação do lobo (*Canis lupus*, L.) no Noroeste de Portugal. 2011, In: PhD Thesis. Universidade de Lisboa, Portugal.

[13] Cabral M.J, Almeida J., Almeida P.R., Dellinger T., Ferrand de Almeida N., Oliveira M., et al Livro vermelho dos vertebrados de Portugal. Instituto da Conservação da Natureza, Lisboa, 2005, pp. 660.

[14] Gazzola A., Bertelli I., Avanzinelli E., Tolosano A., Bertotto P., & Apollonio M. Predation by wolves (*Canis lupus*) on wild and domestic ungulates of the western Alps, Italy. J Zool. 2005, 266(2), pp. 205–213.

[15] Milanesi P., Meriggi A., & Merli E. Selection of wild ungulates by wolves *Canis lupus* (L. 1758) in an area of the Northern Apennines (North Italy). Ethol Ecol Evol. 2012, 24(1), pp. 81–96.

[16] Vos J. Food habits and livestock depredation of two Iberian wolf packs (*Canis lupus signatus*) in the north of Portugal. J Zool. 2000, 251(4), pp. 457–462.

[17] Iliopoulos Y., Sgardelis S., Koutis V., & Savaris D. Wolf depredation on livestock in central Greece. Mammal Res. 2009, 54(1), pp. 11–22.

[18] Nowak S., Mysłajek R. W., & Jędrzejewska B. Patterns of wolf *Canis lupus* predation on wild and domestic ungulates in the Western Carpathian Mountains (S Poland). Acta Theriol. 2005, 50(2), pp. 263–276.

[19] Ansorge H., Kluth G., Hahne S. Feeding ecology of wolves *Canis lupus* returning to Germany, Acta Theriol. 2006, 51 (1) pp. 99–106.

[20] Müller S. Diet composition of wolves (*Canis lupus*) on the Scandinavian peninsula determined by scat analysis. Sweden. 2006, In PhD thesis, Swedish University of Agricultural Sciences, Uppsala, pp. 238 p.

[21] Lanszki J., Márkus M., Újváry D., Szabó Á., & Szemethy L. Diet of wolves *Canis lupus* returning to Hungary, Acta Theriol. 2012, 57(2), pp. 189–193. 10.1007/s13364-011-0063-8 22448046 PMC3294219

[22] Wagner C., Holzapfel M., Kluth G., Reinhardt I., & Ansorge H. Wolf (*Canis lupus*) feeding habits during the first eight years of its occurrence in Germany. Mamm Biol. 2012, 77(3), pp. 196–203.

[23] Ferretti F., Lovari S., Mancino V., Burrini L., & Rossa M. Food habits of wolves and selection of wild ungulates in a prey-rich Mediterranean coastal area. Mamm Biol. 2019, 10.1016/j.mambio.2019.10.008

[24] Papageorgiou N., Vlachos C., Sfougaris A., & Tsachaldis E. Status and diet of wolves in Greece, Acta Theriol. 1994, 39(4), pp. 411–416.

[25] Salvador A., Abad P.L. Food habits of a wolf population (*Canis lupus*) in León province, Spain. Mammalia. 1987, 51, pp. 45–52.

[26] Paixão de Magalhães C. & Petrucci-Fonseca F. The wolf in Bragança county. Impact on cattle and game. Transactions International Congress Game Biologists. 1982, 14, pp. 281–286.

[27] Roque S., Alvares F., & Petrucci-Fonseca F. Utilización espacio-temporal y hábitos alimenticios de un grupo reproductor de lobos en el noroeste de Portugal. Galemys, 13 (special issue). 2001, pp. 179–191.

[28] Vingada J., Fonseca C., Cancela J., Ferreira J., & Eira C. Ungulates and their management in Portugal. European ungulates and their management in the 21st century; 2010 pp. 392–418

[29] Carvalho J., Torres R.T., Acevedo P., Santos J.P.V., Barros T., Serrano, et al (2018). Propagule pressure and land cover changes as main drivers of red and roe deer expansion in mainland Portugal. Diversity and Distributions, 24: 551–564. 10.1111/ddi.12703

[30] Torres R.T., Miranda J., Carvalho J. & Fonseca C. (2015). Expansion and current status of roe deer (*Capreolus capreolus*) on the edge of its distribution, Portugal. Annales Zoologici Fennici, 51: 339–352. 10.5735/086.052.0508

[31] Valente A. M., Fonseca C., Marques T. A., Santos J. P., Rodrigues R., & Torres R. T. Living on the edge: roe deer (*Capreolus capreolus*) density in the margins of its geographical range. PloS one. 2014, 9(2), e88459 10.1371/journal.pone.0088459 24533091 PMC3922805

[32] Torres R. T., Valente A. M., Marques T. A., & Fonseca C. Estimating red deer abundance using the pellet-based distance sampling method. J Forest Sci. 2015, 61, pp. 422–430.

[33] Cuesta L., Barcena F., Palacios F., & Reig S. The trophic ecology of the Iberian wolf (*Canis lupus signatus* Cabrera, 1907). A new analysis of stomach’s data. Mammalia, 1991, 55(2), pp. 239–254.

[34] Barja I. Prey and prey-age preference by the Iberian wolf *Canis lupus signatus* in a multiple-prey ecosystem. Wildlife Biol., 2009, 15(2), pp. 147–155.

[35] Castro J., de Figueiredo T., Fonseca F., Castro J.P., Nobre S., & Pires L.C. Montesinho Natural Park: General description and natural values. Natural heritage from east to west: Case studies from 6 EU countries. New York: Springer 2010, pp. 119–132.

[36] INE, Recenseamento Agrícola 2009—Análise dos principiais resultados. Instituto Nacional de Estatística, I.P. Lisboa (2011).

[37] Saccone C., Attimonelli M., & Sbisa E. Structural elements highly preserved during the evolution of the D-loop-containing region in vertebrate mitochondrial DNA. J Mol Evol. 1987, 26(3), pp. 205–211. 10.1007/bf02099853 3129568

[38] Vilà C., Amorim I. R., Leonard J. A., Posada D., Castroviejo J., Petrucci‐Fonseca, et al Mitochondrial DNA phylogeography and population history of the grey wolf *Canis lupus*. Mol Ecol. 1999, 8(12), pp. 2089–2103. 10.1046/j.1365-294x.1999.00825.x 10632860

[39] Barros T., Gaubert P., Rocha R. G., Bandeira V., Souto L., Mira, et al Mitochondrial demographic history of the Egyptian mongoose (*Herpestes ichneumon*), an expanding carnivore in the Iberian Peninsula. Mamm Biol. 2016, 81(2), pp. 176–184.

[40] Tamura K., Stecher G., Peterson D., Filipski A., & Kumar S. MEGA6: molecular evolutionary genetics analysis version 6.0. Mol Biol Evol. 2013, 30(12), pp. 2725–2729. 10.1093/molbev/mst197 24132122 PMC3840312

[41] Teenrik B. J., 1991 Hair of west European mammals. Cambridge University Press, Cambridge pp. 223.

[42] De Marinis A. M., & Asprea A. Hair identification key of wild and domestic ungulates from southern Europe. Wildlife Biol. 2006, 12(3), pp. 305–320.

[43] Valente A. M., Rocha R. G, Delgado E, Ferreira J. P. e Fonseca C. Atlas dos Pelos dos Mamíferos Terrestres Ibéricos. In: Edições Afrontamento. (EDS.), 9789723614510; 2015.

[44] Floyd T. J., Mech L. D., & Jordan P. A. Relating wolf scat content to prey consumed. J Wild Manage. 1978, pp. 528–532.

[45] Ciucci P., Boitani L., Pelliccioni E. R., Rocco M., & Guy I. A comparison of scat-analysis methods to assess the diet of the wolf *Canis lupus*. Wildlife Biol. 1996, 2(1), pp. 37–48.

[46] Ruprecht A. L. Food of the Barn owl, *Tyto alba guttata* (CL Br.) from Kujawy. Acta Ornithol. 1979, 15(19), pp. 493–512.

[47] R Core Team 2019. R: A language and environment for statistical computing. R Foundation for Statistical Computing, Vienna, Austria URL https://www.R-project.org/.

[48] Weaver J. L. Refining the equation for interpreting prey occurrence in gray wolf scats. J Wild Manage. 1993, pp. 534–538.

[49] DRAEDM. Guia para a identificação de raças caprinas e ovinas no Entre Douro e Minho. Formação Profissional Agrária, 1993, 20. pp. 54.

[50] MacDonald, D. W. & Barret, P. Mammals of Britain and Europe. (Eds.). Collins Field Guide Series, Harper Collins Publishers, Great Britain. 1993, 312 pp.

[51] Llaneza L. U. I. S., Fernández A., & Nores C. Dieta del lobo en dos zonas de Asturias (España) que difieren en carga ganadera. Doñana, Acta Vertebrata. 1996, 23(2), pp. 201–213.

[52] Sarmento P. Feeding ecology of the European wildcat *Felis silvestris* in Portugal. Acta Theriol. 1996, 41(4), pp. 409–414.

[53] Menon V. Indian mammals: a field guide. Hachette India. Book Publishing India Pvt. Ltd 2014, pp. 528.

[54] Roy S., Ghoshal A., Bijoor A., & Suryawanshi K. Distribution and activity pattern of stone marten *Martes foina* in relation to prey and predators. Mammal Biol. 2018 10.1016/j.mambio.2018.09.013

[55] Levins R. Evolution in changing environments: some theoretical explorations (No. 2). Princeton University Press 1968.

[56] Hurlbert SH. The measurement of niche overlap and some relatives. Ecology. 1978, 59: pp. 67–77.

[57] Weaver W, Shannon CE. The mathematical theory of communication. Illinois University Press, Urbana, Illinois 1949.

[58] Vogel J. T., Somers M. J., & Venter J. A. The foraging ecology of reintroduced African wild dog in small protected areas. Wildlife Biol. 2018, wlb-00424.

[59] Ivlev V. S. Experimental ecology of the feeding of fishes. New Haven: Yale Univ. Press. 1961.

[60] Jacobs J. Quantitative measurement of food selection. Oecologia. 1974, 14(4), pp. 413–417. 10.1007/BF00384581 28308662

[61] Lagos L. & Bárcena F. Spatial variability in wolf diet and prey selection in Galicia (NW Spain). Mammal Res, 2018, 63, pp. 125–139

[62] Salazar D.C. Distribuição e estatuto do veado e corço em Portugal. Doctoral dissertation, University of Aveiro 2009 Available from: https://ria.ua.pt/bitstream/10773/854/1/2009001236.pdf.

[63] Andersone Ž., & Ozoliņš J. Food habits of wolves *Canis lupus* in Latvia. Acta Theriol. 2004, 49(3), pp. 357–367.

[64] Mateos-Quesada P. & Carranza J. Reproductive patterns of roe deer in central Spain. Etología, 2000, 8, pp. 17–20.

[65] Palumbo, D. I lupi del parco del Corno alle Scale. Bolognia, Riccrche e monotoraggi sulla presenza dell lupo nell’Appennino Bolognese. 2003, p. 52 (In Italian)

[66] Jędrzejewski W., Niedziałkowska M., Hayward M. W., Goszczyński J., Jędrzejewska B., Borowik, et al Prey choice and diet of wolves related to ungulate communities and wolf subpopulations in Poland. J Mammal. 2012, 93(6), pp. 1480–1492.

[67] Huggard DJ. Prey selectivity of wolves in Banff National Park. I. Prey species. Can J Zool. 1993, 71: pp. 130–139.

[68] Okarma H. The trophic ecology of wolves and their predatory role in ungulate communities of forest ecosystems in Europe. Acta Theriol. 1995,40(4), pp. 335–386.

[69] Nores C., Llaneza L., & Álvarez Á. Wild boar *Sus scrofa* mortality by hunting and wolf *Canis lupus* predation: an example in northern Spain. Wildlife Biol. 2008, 14(1), pp. 44–51.

[70] Leonard J. A. Ecology drives evolution in grey wolves. Evol Ecol Res. 2015, 16(6), pp. 461–473.

[71] Pimenta V., Barroso I., Boitani L., & Beja P. Wolf predation on cattle in Portugal: Assessing the effects of husbandry systems. Biol Conserv. 2017, 207, pp. 17–26.

[72] Røskaft E., Händel B., Bjerke T., & Kaltenborn B. P. Human attitudes towards large carnivores in Norway. Wildlife Biol. 2007, 13(2), pp. 172–185.

[73] Pimenta V., Barroso I., Boitani L., & Beja P. Risks a la carte: Modelling the occurrence and intensity of wolf predation on multiple livestock species. Biol Conserv. 2018 228, pp. 331–342.

[74] Ballard W.B.; Carbyn L.N. & Smith D.W. Wolf interactions with non-prey. In: Mech L.D.& Boitani L. (Eds). Wolves; Behavior, Ecology and Conservation The University of Chicago Press, Chicago 2003, pp. 259–271.

