# Supplementary material for: What does the wolf eat? Assessing the diet of the endangered Iberian wolf (Canis lupus signatus) in northeast Portugal
Source: PLoS One. 2020 Mar 31;15(3):e0230433. doi: 10.1371/journal.pone.0230433 (PMC7108738; doi:10.1371/journal.pone.0230433)
Supplement: S1 File — (DOCX) [file pone.0230433.s001.docx]

| Scat code | Wild boar | Red deer | Roe deer | Domestic Goat | Domestic/wild cat | Stone marten | Small mammals |
| --- | --- | --- | --- | --- | --- | --- | --- |
| L1 | X |  |  |  |  |  |  |
| L2 |  |  | X |  |  |  |  |
| L3 |  | X | X |  |  |  |  |
| L4 |  |  |  |  |  | X |  |
| L5 |  |  |  | X |  | X |  |
| L6 |  | X |  |  |  |  |  |
| L7 |  |  | X |  |  |  |  |
| L8 |  |  | X |  |  |  |  |
| L9 |  |  | X |  |  |  |  |
| L10 | X |  |  |  |  |  |  |
| L11 |  | X |  |  |  |  |  |
| L12 |  |  | X |  |  |  |  |
| L13 |  | X |  |  |  |  |  |
| L14 | X |  |  |  |  |  |  |
| L15 |  |  | X |  |  |  |  |
| L16 |  |  | X |  |  |  |  |
| L17 |  | X |  |  |  |  |  |
| L18 |  | X |  |  |  |  |  |
| L19 | X | X |  |  |  |  |  |
| L20 |  |  | X |  |  | X |  |
| L21 |  | X |  |  |  |  |  |
| L22 |  |  | X |  |  |  |  |
| L23 |  |  |  |  | X |  | X |
| L24 | X |  |  |  |  |  |  |
| L25 | X |  |  |  |  |  |  |
| L26 | X |  |  |  |  |  |  |
| L27 | X |  |  |  |  |  |  |
| L28 | X |  |  |  |  |  |  |
| L29 |  | X |  |  |  |  |  |
| L30 | X |  |  |  |  |  |  |
| L31 | X |  |  |  |  |  |  |
| L32 |  | X |  |  |  |  |  |
| L33 | X |  |  |  |  |  |  |
| L34 |  |  | X |  |  |  |  |
| L35 |  | X |  |  |  |  |  |
| L36 | X |  |  |  |  |  |  |
| L37 |  | X |  |  |  |  |  |
| L38 |  |  | X |  |  |  |  |
| L39 | X |  |  |  |  |  |  |
| L40 | X |  |  |  |  |  |  |
| L41 |  |  |  |  |  |  | X |
| L42 |  |  | X |  |  |  |  |
| L43 |  | X |  |  |  |  |  |
| L44 |  |  | X |  |  |  |  |
| L45 | X |  |  |  |  |  |  |
| L46 |  |  |  |  | X |  |  |
| L47 |  | X |  |  |  |  |  |
| L48 |  | X |  |  | X |  |  |
| L49 |  |  |  | X |  |  |  |
| L50 |  |  |  | X |  |  |  |
| L51 |  |  | X |  |  |  |  |
| L52 |  |  | X |  |  |  |  |
| L53 | X |  |  |  |  |  |  |
| L54 |  | X |  |  |  | X |  |
| L55 |  |  |  |  |  |  | X |
| L56 |  | X |  |  |  |  |  |
| L57 |  | X |  |  |  |  |  |
| L58 |  |  | X |  |  |  |  |
| L59 |  | X |  |  |  |  |  |
| L60 |  |  | X |  |  |  |  |
| L61 |  |  | X |  |  |  |  |
| L62 |  |  | X |  |  |  |  |
| L63 |  | X | X |  |  |  |  |
| L64 |  |  | X |  |  |  |  |
| L65 |  |  | X |  |  |  |  |
| L66 |  |  |  |  | X |  |  |
| L67 |  |  | X |  |  |  |  |
| L68 |  |  | X | X |  |  |  |
| L69 |  |  | X |  |  |  |  |
| L70 |  |  | X |  |  |  |  |
| L71 |  |  | X |  |  |  |  |
| L72 |  |  | X |  |  |  |  |
| L73 |  |  | X |  |  |  |  |
| L74 |  | X |  |  |  |  |  |
| L75 | X |  |  |  |  |  |  |
| L76 |  |  | X |  |  |  |  |
| L77 |  |  | X |  |  |  |  |
| L78 |  |  | X |  | X |  |  |
| L79 | X |  |  |  |  |  |  |
| L80 |  | X |  |  |  |  |  |
| L81 |  |  | X |  |  |  |  |
| L82 |  |  | X |  |  |  |  |
| L83 | X |  | X |  |  |  |  |
| L84 |  |  | X |  |  |  |  |
| L85 |  | X |  |  |  |  |  |
